# CD38-targeted therapy with Daratumumab in clinical lung transplantation: A single-center experience

**DOI:** 10.1016/j.jhlto.2025.100386

**Published:** 2025-09-16

**Authors:** Caroline Hillebrand, Giuseppe Maggioni, Sophia Auner, Panja Maria Boehm, Daniela Koren, Zsofia Kovacs, Stefan Schwarz, Gottfried Fischer, Antonia Müller, Renate Kain, Konrad Hoetzenecker, Clemens Aigner, Fiorella Calabrese, Peter Jaksch, Alberto Benazzo

**Affiliations:** a Department of Thoracic Surgery, Medical University of Vienna, Vienna, Austria; b Department of Cardiac, Thoracic and Vascular Sciences, University of Padua, Padua, Italy; c Department of Pathology, Medical University of Vienna, Vienna, Austria; d Department of Blood Group Serology and Transfusion Medicine, Medical University of Vienna, Vienna, Austria; e Comprehensive Center for Chest Diseases, Medical University of Vienna, Vienna, Austria; f Department of Thoracic Surgery, Vanderbilt University Medical Center, Nashville, TN

**Keywords:** Lung transplantation, Antibody-mediated rejection, Daratumumab, CD38

## Abstract

**Background:**

Antibody-mediated rejection remains a major threat to long-term graft function after lung transplantation. Current therapies aim to eliminate circulating antibodies and suppress B-cell-activity but often fail to reduce donor-specific antibodies. Daratumumab, a monoclonal antibody targeting CD38, has shown potential in depleting antibody-producing plasma cells. This study investigates the clinical application of daratumumab in lung transplant recipients.

**Methods:**

We performed a retrospective single-center study including all lung transplant recipients treated with subcutaneous daratumumab for antibody-mediated rejection A total of 14 patients with newly developed donor-specific antibodies and clinical antibody-mediated rejection were analyzed.

**Results:**

In all patients with AMR, antibodies directed against human leukocyte antigen class I decreased to less than 25–50% of baseline levels within 12 weeks. Antibodies against class II also declined in 5 patients. Eleven patients survived the initial AMR episode. Chronic lung allograft dysfunction was already present in several patients before the AMR episode, while others developed CLAD during follow-up. The treatment was generally well tolerated with the most common side effects being leukopenia, hypogammaglobulinemia and infections.

**Conclusions:**

CD38-targeted therapy with daratumumab may represent a promising addition to the antibody mediated rejection treatment panel.

## Background

Antibody-mediated rejection (AMR) after lung transplantation is a major contributor to early graft loss and chronic lung allograft dysfunction (CLAD). It is primarily driven by donor-specific antibodies (DSA), typically directed against human leukocyte antigen (HLA) class I or II, which cause acute and chronic allograft injury.^1^ A particularly vulnerable subgroup consists of sensitized patients with preformed HLA antibodies, who are at increased risk for AMR. To address this, various desensitization strategies have been developed.^2^, ^3^

Data on AMR management in lung transplantation remain limited. Current therapeutic approaches fall into three categories: unspecific antibody elimination (e.g. plasma exchange), B cell or plasma cell depletion (e.g. anti-CD20 antibodies, proteasome inhibitors) and complement inhibition. Despite their use, no strategy has consistently demonstrated favorable outcomes in lung transplant recipient.^4^

CD38 is a transmembrane glycoprotein highly expressed on plasma cells and natural killer (NK) cells. Daratumumab, a monoclonal antibody targeting CD38, is approved for multiple myeloma treatment.^5^ In AMR, daratumumab may offer benefits by depleting plasma cells and suppressing NK cells, both key effectors in the humoral alloimmune response.^6^ Based on encouraging data in kidney and heart transplantation,^6^, ^7^, ^8^, ^9^, ^10^, ^11^ we initiated daratumumab treatment in selected lung transplant recipients with severe AMR.

## Methods

This retrospective single-center study included all patients who received daratumumab as adjunctive therapy for AMR at the Department of Thoracic Surgery at the Medical University of Vienna. Data on baseline demographics, immunological characteristics and outcomes were analyzed. Follow-up was conducted until April 30, 2024. The study was approved by the institutional review board of the Medical University of Vienna (EK-Nr 1984/2022). Informed consent was waived due to the retrospective nature of the study. The study was conducted in accordance with the Declaration of Helsinki and the ISHLT Ethics Statement. Due to the exploratory nature and small sample size, findings should be interpreted as preliminary and informative for future prospective investigations.

### Clinical protocol and definitions

Post-transplant immunosuppression followed institutional standards. Patients received either a single 30 mg dose of alemtuzumab (Genzyme/Sanofi, Cambridge, USA) or no induction. After alemtuzumab, low-dose tacrolimus and steroids were administered during the first year, with mycophenolate added thereafter. If no induction was given, tacrolimus was started at a higher level with immediate mycophenolate initiation.^12^, ^13^

Perioperative antimicrobial prophylaxis included broad-spectrum antibiotics, adapted based on donor and recipient colonization. Antifungal prophylaxis consisted of inhaled amphotericin B for three months. Pneumocystis jirovecii prophylaxis with trimethoprim- sulfamethoxazole was given lifelong. Cytomegalovirus (CMV) prophylaxis included valganciclovir and CMV-IVIG (Cytotect; Biotest GmbH, Vienna, Austria) for 3 months (or 12 months in high-risk mismatch, D+/R-).

Surveillance bronchoscopies with transbronchial biopsies were scheduled at 2 weeks, 1, 2, 3, 6 and 12 months. Spirometry with plethysmography and DSA testing occurred at the same intervals and during episodes of clinical decline.

Diagnosis of CLAD followed ISHLT criteria.^14^ Infections were defined by clinical signs and need for treatment. Clinical response to daratumumab was assessed retrospectively, based on a combination of (1) reduction in DSA, (2) stabilization or improvement of lung function, (3) absence of further treatment escalation and/or (4) recovery from respiratory insufficiency.

### AMR treatment

AMR therapy typically began with immunoadsorption (IA). It should be noted, however, that due to the absence of a standardized treatment protocol, therapy varied among patients. For IA, plasma was separated by centrifugation (COBE Spectra® or Spectra OPTIA® Apheresis System; Terumo BCT) under anticoagulation with citrate and heparin. Each IA session treated 2.5 plasma volumes.

In case of insufficient response, anti-thymocyte globulin (ATG, 2mg/kg for 5 days) and intravenous immunoglobulin (IVIG, Privigen® [CSL Behring, Germany], 1–2 g/kg daily for 2–5 consecutive days) were added. If AMR persisted or DSA levels remained high, daratumumab was given subcutaneously at 1800 mg weekly. The number of doses varied depending on individual clinical response and tolerability. Therapy was continued until patients showed clinical improvement or discontinued in case of relevant complications such as infections, leukopenia or elevated CRP. Acute AMR patients often received fewer doses due to early infectious complications. No additional AMR treatments were given after daratumumab.

### HLA antibody detection

DSA were assessed using LABScreen® Single Antigen Beads (One Lambda, Canoga Park, CA, USA) on the Luminex FlexMap 3D platform (Luminex Corporation, Austin, TX, USA). If MFI exceeded 20.000, samples were diluted and results multiplied by the dilution factor. MFI > 1000 was considered positive. Measurements were analyzed using the HLA Fusion software (Thermo Fisher Scientific Inc.). DSA were categorized as: Category I (MFI 1000–2000), Category II (MFI 2000–5000), Category III (MFI 5000–10,000), Category IV (MFI > 10,000).

### Morphological evaluation (histology and immunohistochemistry)

Transbronchial biopsies were evaluated according to the Lung Allograft Standardized Histological Analysis (LASHA).^15^ Immunohistochemistry (IHC) was performed for CD38 (clone SPC32; Leica Microsystems), CD57 (clone NK-1; Leica Microsystems), C4d (clone A24-T; DB Biotech) and ph-S6RP (Ser235/236; clone D57.2.2E; Euroclone). AMR surrogates (C4d and ph-S6RP) were scored as described previously.^16^ CD38 and CD57 staining was performed by two blinded pathologists using a semiquantitative 4-point scoring system (0: no staining; 1: focal staining, <10% of inflammatory infiltrate; 2: multifocal staining, 11%−30% of inflammatory infiltrate; 3: diffuse staining, >30% of inflammatory infiltrate).

### Statistical analysis

Categorical variables were expressed as total numbers and percentages. Continuous variables were presented as medians (interquartile range) or means (± SD). Data was analyzed using IBM SPSS Statistics 29 (Version 29.0.1.0) and figures were designed with GraphPad Prism version 9 (GraphPad Software, San Diego, CA). Due to the retrospective design and limited sample size, no inferential statistical tests were performed. Date was analyzed descriptively to explore trends in clinical response and immunological markers following daratumumab therapy.

## Results

Between January 2018 and April 2023, 14 patients received daratumumab as treatment for AMR at our center. 537 lung transplants were performed during this period. The majority of patients receiving daratumumab was female (9/14; 64.3%), with a median age of 48 years (IQR: 32–50). The predominant indication was restrictive lung disease (8/14; 57.1%), followed by obstructive lung conditions (5/14; 35.7%).

Eight patients (57.1%) developed de novo DSA (dnDSA) after transplantation, while six (42.9%) were pre-sensitized with increased post-transplant DSA. Twelve (85.7%) received alemtuzumab induction; two did not receive induction (one with cystic fibrosis and Burkholdera colonization, one transplanted for Covid19-ARDS). The median time from transplant to dnDSA detection (MFI > 1000) was 19 days (IQR: 12.5–332). AMR was diagnosed at 307 days (IQR: 105.3–437.5) post-transplant in dnDSA patients and 9 days (IQR: 8–129) in pre-sensitized cases. In these early cases, AMR was distinguished from primary graft dysfunction (PGF) by presence of DSA, lack of response to PGD therapy and histological/immunohistochemical findings.

Most patients (11/14; 78.6%) had DSA against both HLA class I and II; three had class II DSA only. Class I DSA were mainly directed against HLA-B (n=11; 78.6%), followed by HLA-A (n=6; 42.9%) and HLA-C (n=5; 35.7%). Among class II antigens, all patients had DSA directed against HLA-DQ (n=14; 100%), followed by antibodies against HLA-DR (n=10; 71.4%) and HLA-DP (n=4; 28.6%).

Two patients (14.3%) met criteria for definite AMR, seven (50%) for probably and five (35.7%) for possible AMR. Detailed patient characteristics are summarized in Table 1.

**Table 1:** Patient Characteristics for Patients with AMR

Patient Characteristics (AMR Group)		
		n=14
Female		9 (64.3)
Age at Tx (years), median (IQR)		48 (32−59)
Primary Disease	Obstructive, No. (%)	5 (35.7)
	Restrictive, No. (%)	8 (57.1)
	Others, No. (%)	1 (7.1)
AMR Type	AMR (de novo DSA), No. (%)	8 (57.1)
	AMR (pre-sensitized), No. (%)	6 (42.9)
Grading of AMR	Definite, No. (%)	2 (14.3)
	Probable, No. (%)	7 (50.0)
	Possible, No. (%)	5 (35.7)
Presensitization*	HLA class I only, No. (%)	1 (16.7)
	HLA class II only, No. (%)	3 (50.0)
	HLA class I and II, No. (%)	2 (33.3)
Tx Type	Double-lung, No. (%)	13 (92.6)
	Single-lung, No. (%)	1 (7.1)
Induction therapy	Alemtuzumab, No. (%)	12 (85.7)
	None, No. (%)	2 (14.3)
Time between Tx and post-transplant DSA (MFI > 1000) in days**, median (IQR)		21 (13−345)
Time between post-transplant DSA and AMR Diagnosis in days**, median (IQR)		90 (0−386)
Time between Tx and AMR Diagnosis	AMR (de novo DSA) (days), median (IQR)	333 (221−440)
	AMR (pre-sensitized) (days), median (IQR)	12 (9−124)
Post-transplant DSA Class	HLA class I only, No. (%)	0
	HLA class II only, No. (%)	3 (21.4)
	HLA class I and II, No. (%)	11 (78.6)
Post-transplant DSA highest MFI	Category I (MFI 1000−2000), No. (%)	0
	Category II (MFI 2000−5000), No. (%)	0
	Category III (MFI 5000−10.000), No. (%)	2 (14.3)
	Category IV (MFI > 10.000), No. (%)	12 (85.7)
High-grade ACR, No. (%)		1 (7.1)
High-grade LB, No. (%)		0 (0)
Diffuse Alveolar Damage (DAD)***, No. (%)		9 (64.3)
Inflammatory Capillaritis		10 (71.5)
Septal Widening		8 (57.1)
ph-S6RP (score = 3)		5 (35.7)
C4d Positivity, No. (%)		2 (14.3)
Positive cell-free DNA, No. (%)		10 (71.4)

* for pre-sensitized patients (n=6)

** for patients with de novo DSA (n=8)

*** according to LASHA evaluation template DAD includes (1) Hyaline membranes, (2a) septal and (2b) intra-alveolar organizing pneumonia (OP)

Eight patients (57.1%) required ICU admission for respiratory failure. Prior to the administration of daratumumab, all patients had undergone various AMR therapy therapies (see Table 2). Three patients (21.4%) received a combination of ATG, immunoadsorption and plasmapheresis, three (21.4%) received ATG and immunoadsorption and another three (21.4%) received ATG and plasmapheresis. Five patients (35.7%) were treated with immunoadsorption alone. Median time from AMR diagnosis to first daratumumab dose was 13 days (IQR: 5–45). The total number of doses administered ranged from 2 to 9.

**Table 2:** AMR Treatment

AMR Treatment			
			n=14
ICU admission			8 (57.1)
ATG + Immunoadsorption + Plasmapheresis, No. (%)			3 (21.4)
ATG + Immunoadsorption, No. (%)			3 (21.4)
ATG + Plasmapheresis, No. (%)			3 (21.4)
Immunoadsorption, No. (%)			5 (35.7)
Time from AMR diagnosis to first dose of daratumumab in days, median (IQR)			13 (5−45)
Number of doses of daratumumab, median (IQR)			3 (2−5)
Side Effects	Leukopenia, No. (%)		7 (50.0)
	Hypogammaglobulinemia (IgG < 600mg/dl)*, No. (%)		12 (85.7)
	Infection, No. (%)	Total, No. (%)	9 (64.2)
		Bacterial, No. (%)	8 (57.1)
		Viral, No. (%)	1 (7.1)
		Fungal, No. (%)	3 (21.4)

* baseline IgG levels prior to daratumumab administration were available in 9 patients

Hypogammaglobulinemia (IgG < 600 mg/dl) occurred in 12/14 (85.7%) patients. Eight of these required administration of IVIG. Baseline IgG values prior to daratumumab administration were available in 9 patients; 1 already had hypogammaglobulinemia, while the remaining 8 showed normal levels. 7 patients (50%) developed leukopenia. Infections were observed in 9 patients (64.2%): 8 bacterial, 1 viral, 3 fungal. Nine patients (64.3%) developed CMV DNAemia during daratumumab therapy. In three cases, low-level DNAemia was already present prior to treatment and subsequently increased, while six patients developed new-onset DNAemia consistent with reactivation. No cases of primary CMV infection were observed.

**Figure 1:**
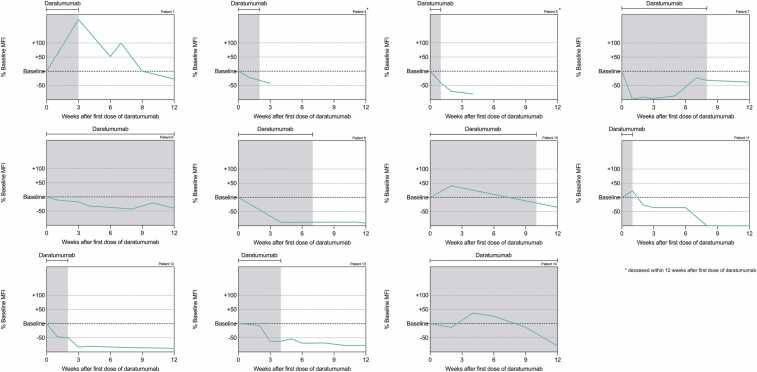
**Development of DSA against HLA class I after administration of daratumumab in patients with AMR.** Each panel represents the trajectory of percentage change in MFI of HLA class I antibodies relative to baseline over a 12-week period starting from the first dose of daratumumab. The shaded area indicates the treatment period with daratumumab. Data is shown for 11 of the 14 patients treated; patients 2, 3 and 6 were excluded because they did not have detectable DSA against HLA class I.

**Figure 2:**
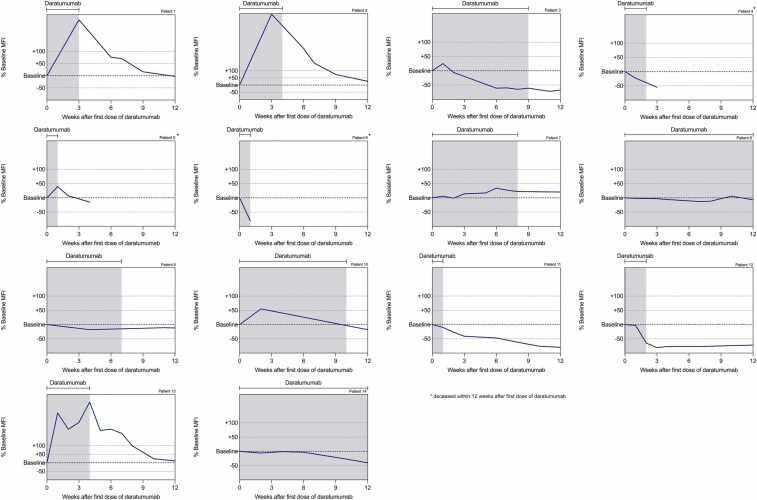
**Development of DSA against HLA class II after administration of daratumumab in patients with AMR.** Each panel represents the trajectory of percentage change in MFI of HLA class II antibodies relative to baseline over a 12-week period starting from the first dose of daratumumab. The shaded area indicates the treatment period with daratumumab.

Figure 1, Figure 2 show the trajectory of DSA following the administration of daratumumab in the 14 patients with AMR. DSA against HLA class I declined to < 25% of baseline within 12 weeks in all patients; 6 patients dropped to < 50%. DSA against class II decreased to < 50% in 5 patients and increased in 3. Of all patients, 11 (78.6%) survived the early phase following AMR treatment. CLAD was diagnosed in 7 patients (50%). In 5 cases, the diagnosis had already been established before daratumumab treatment, while in 2 patients CLAD developed after therapy at 74 and 477 days following the last daratumumab dose. Three patients subsequently underwent retransplantation. Median time from AMR diagnosis to death was 225 days (IQR: 53–678) and to retransplantation 194 days (IQR: 182–206). Outcome parameters are summarized in Table 3.

**Table 3:** Outcome of Patients with AMR

Outcome (AMR Group)		
		n=14
Acute AMR survival followed by hospital discharge, No. (%)		11 (78.6)
6-month survival, No. (%)		11 (78.6)
1-year survival, No. (%)		9 (64.3)
CLAD present before or at AMR diagnosis, No. (%)		5 (35.7)
Diagnosis of CLAD after AMR diagnosis, No. (%)		2 (14.3)
CLAD Type	Bronchiolitis obliterans syndrome, No. (%)	4 (28.6)
	Restrictive allograft syndrome, No. (%)	0 (0.0)
	Mixed, No. (%)	3 (21.4)
Re-Transplant, No. (%)		3 (21.4)
Time between AMR Diagnosis and Re-Transplant in days, median (IQR)		194 (182−206)
Time between AMR Diagnosis and Death in days, median (IQR)		225 (53−678)

A granular evaluation of morphological parameters according to the LASHA evaluation template detected histological lesions evocative of AMR in all cases except one (13/14; 93%). In particular, the presence of different degrees of inflammatory capillaritis was detected in 10/14 (71.5%), with only 1 of them showing the most severe form, neutrophilic capillaritis; the presence of severe edema with widening was detected in 8/14 (57.1%) patients, and lastly, diffuse alveolar damage (DAD), which includes septal organizing pneumonia (OP) and/or hyaline membranes and pneumocyte hypertrophy) was detected in 9/14 (64.3%) patients.

C4d was positive in 2/14 (14.3%) cases. Ph-S6RP (score < 2) was found in in 9/14 cases (64.3%). It was strongly expressed (score = 3) in 5/14 cases (35.7%), thereby surpassing C4d in sensitivity for detecting AMR.

CD57 and CD38 (score > 1) were detected in 8 and 10 patients, respectively. The rest could not be assessed due to inconsistent biopsy size and sampling area adequacy. Among the interpretable biopsies, the mean score for CD57 and CD38 (in inflammatory cells) before treatment was 0.71 and 1.15, respectively.

**Figure 3:**
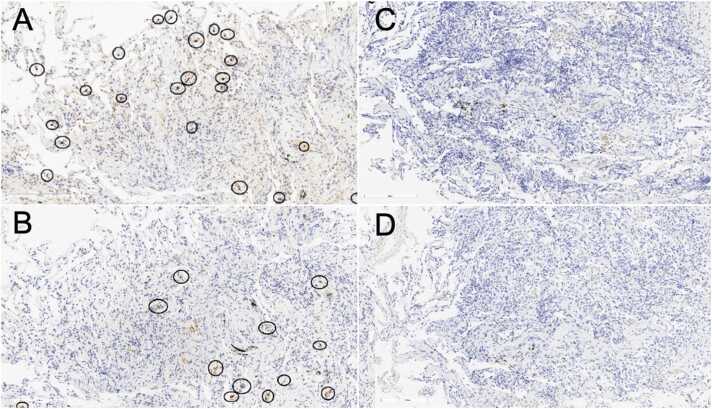
**Image panel of immunohistochemistry stainings CD38 and CD57 before and after treatment.** Panel figure from a patient with probable AMR (C4d negative) treated with daratumumab and biopsied before and 4 months after the treatment. A: CD57 before treatment (score 2); B: CD38 before treatment (score 2); C: CD57 after treatment (score 0); D: CD38 after treatment (score 0).

Three cases of biopsies performed after treatment showed a notable reduction of CD38 and CD57 positive cells in the inflammatory infiltrate. A panel image is provided as Figure 3, where a significant clearance of CD38 and CD57 positive cells after drug administration is shown.

## Discussion

This descriptive case series provides initial insight into the potential role of daratumumab as an add-on therapy for refractory AMR in clinical lung transplantation. The primary aim was to assess daratumumab’s efficacy in reducing DSA and to explore its impact on patient and graft survival. In most AMR cases, daratumumab successfully decreased DSA, particularly against HLA class I, representing a promising rescue option. However, the main side effects were hypogammaglobulinemia, leukopenia and infections, warranting caution and further investigation.

The role of DSA in AMR pathogenesis is well established: DSA bind to HLA on the allograft’s endothelium and activate immune cascades.^17^ Moreover, sensitization prior to transplantation increase AMR risk and delays access to grafts.^2^ Experience with daratumumab in solid organ transplantation remains limited. While isolated reports describe its use in lung transplantation,^18^, ^19^ our study provides the first systematic evaluation of its application for AMR treatment in a clinical cohort. Our findings support earlier kidney and heart transplant data showing MFI reductions of circulating DSA, especially HLA class I.^6^, ^8^, ^9^, ^10^, ^11^ Interestingly, some patients with minimal DSA decrease still had a favorable clinical response, underscored by the fact that more than two thirds of the herein reported patients overcame the initial phase of AMR without early mortality. This observation might suggest that daratumumab exerts mechanisms of action, apart from its effects on DSA, that contribute to its clinical efficiency. On the other hand, some patients showed a reduction in their DSA titers but did not recover and ultimately deceased and/or developed CLAD. This may be explained, at least in part, by the timing of intervention. In our cohort, daratumumab was predominantly used as a last-line rescue therapy, often after multiple prior immunomodulatory strategies had failed. During this interval, ongoing allograft damage may have occurred, reducing the potential for recovery. Thus, the earlier use of daratumumab may enhance therapeutic efficacy.

Recent studies have highlighted the role of daratumumab in targeting natural killer (NK) cells, which also express high levels of CD38.^6^, ^20^, ^21^ NK cells have gained increasing recognition as contributors in the pathogenesis of AMR, particularly through their role in antibody-dependent cellular toxicity (ADCC). In kidney transplant recipients, NK cells contribute to graft rejection by inducing vascular injury, primarily through an activation by FcγRIIIa (CD16) in response to DSA, amplifying the inflammatory and cytotoxic response.^6^, ^20^ Daratumumab has been shown to be efficient in depleting CD38-expressing plasma cells responsible for DSA production. However, it also leads to a significant reduction in circulating and graft-associated NK cells.^6^ This observation is crucial when considering daratumumab’s role in treating AMR, as it suggests a potential therapeutic effect beyond simple plasma cell depletion. For instance, while studies in kidney transplant patients have indicated that daratumumab does not significantly deplete plasma cells in all cases, its ability to reduce NK cell infiltration may offer a novel mechanism to mitigate the vascular inflammation seen in AMR.

The majority of transbronchial biopsies showed indicative AMR lesions, as indicated by using the LASHA template evaluation. AMR surrogate markers were positive in most cases and a significant inflammatory cell infiltration by CD38- and CD57-positive cells was observed. In a subset of patients with evaluable transbronchial biopsies before and after daratumumab treatment, we noted a reduction in CD38- and CD57-positive cells following therapy. These findings suggest a potential histological correlate to the drug’s immunomodulatory effect and support the hypothesis that these markers may serve as theragnostic targets in AMR. However, due to limitations in biopsy availability, inconsistent sampling sites and variable timing relative to therapy a systematic evaluation was feasible in only a limited number of cases. Complete immunostainings from adequate biopsy samples at standardized time points will be essential to validate the utility of CD38 and CD57 as biomarkers of treatment response.

Despite clinical improvement in many AMR patients, long-term outcomes remain poor.^1^ This underscores the severe nature of AMR and the importance of effective intervention strategies. Within our cohort, CLAD was already present in several patients at or before the AMR episode, while others developed CLAD during follow-up, reflecting that daratumumab was predominantly administered as late salvage therapy. Consequently, our data cannot provide conclusions on daratumumab’s effect in preventing CLAD. It is also important to notice that all therapeutic options before the administration of daratumumab had failed, prolonging the period during which the allograft was exposed to ongoing damage. Approaches with earlier use of daratumumab should therefore be evaluated.

Side effects in our cohort were notable. Infections, leukopenia and hypogammaglobulinemia were common, consistent with data from multiple myeloma trials.^22^, ^23^, ^24^ CMV reactivation was frequent in our cohort, consistent with the known increased risk of viral reactivation und B-cell-depleting therapies. No primary infections were documented. In the study by Lokhorst et al.,^22^ grade 3 or 4 adverse events included infections and hematologic toxicities, including neutropenia and thrombocytopenia, further indicating that daratumumab, while being effective, carries significant risks in heavily pretreated populations. In our cohort, baseline immunoglobulin levels prior to daratumumab administration were available for a subset of patients and in at least one case hypogammaglobulinemia was already present before daratumumab administration. Thus, not all cases observed during follow-up can be attributed solely to daratumumab, which precludes a clear distinction between pre-existing hypogammaglobulinemia and treatment-induced IgG depletion. Furthermore, nearly all patients had previously received alemtuzumab and most were treated with ATG, both of which may have contributed to B cell and plasma cell suppression. Thus, it is not clear if the observed events reflect the cumulative immunosuppressive burden or the isolated effect of daratumumab. Given the high level of immunosuppression in lung transplant recipients, it is crucial to implement strategies for thorough infectious prophylaxis, regular immunoglobulin monitoring and timely IVIG substitution during daratumumab treatment.

This study has several important limitations. First, the retrospective case-series design and small sample size preclude statistical inference and limit the generalizability of our findings. Second, the heterogeneous clinical context, including variability in induction therapy, prior AMR treatments and differences in desensitization strategies, introduces substantial confounding. The use of daratumumab as rescue therapy, typically at a late stage when the allograft was already compromised, may have further limited its observed efficacy. Furthermore, the number of daratumumab doses varied among patients, complicating interindividual comparisons. Finally, adequate transbronchial biopsies were not consistently available at standardized time points due to the critical condition of many patients, limiting the systematic histological assessment. However, given the novelty of CD38 targeting in lung transplantation, these data offer valuable first clinical observations to guide hypothesis-driven research.

In conclusion, our findings provide preliminary evidence supporting the use of CD38 targeting with daratumumab in the treatment of AMR in lung transplantation. Future prospective studies with standardized timing, dosing and response assessment will be essential to define the role of daratumumab in AMR treatment algorithms.

## Financial Disclosure Statement

The authors of this manuscript declare that they have no relevant financial or non-financial conflicts of interest related to the content of this manuscript.

## Data Availability Statement

The data that supports the findings of this study are available from the corresponding author upon reasonable request. All data have been anonymized in accordance with ethical guidelines and no identifying information has been included in this manuscript.

## Declaration of Competing Interest

The authors declare that they have no known competing financial interests or personal relationships that could have appeared to influence the work reported in this paper.
